# Supplementary material for: Identification of photoperiod-induced specific miRNAs in the adrenal glands of Sunite sheep (Ovis aries)
Source: Front Vet Sci. 2022 Jul 22;9:888207. doi: 10.3389/fvets.2022.888207 (PMC9354845; doi:10.3389/fvets.2022.888207)
Supplement: Supplementary file 1 [file Data_Sheet_1.ZIP › Supplementary file/miRDeep2 file.zip!/miRDeep2 file/Novel_Structure/LP42A1/Novel_530.pdf]

Provisional ID	:	NC_019466.2_16339
Score total	:	82.8
Score for star read(s)	:	3.9
Score for read counts	:	75.6
Score for mfe	:	1.7
Score for randfold	:	1.6
Score for cons. seed	:	
Total read count	:	160
Mature read count	:	145
Loop read count	:	0
Star read count	:	15

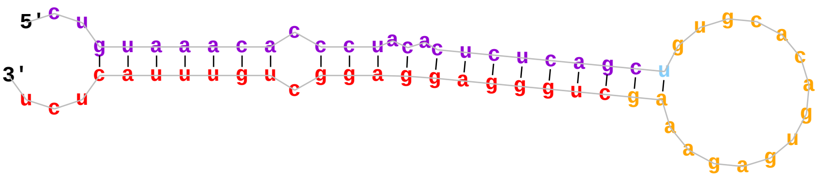

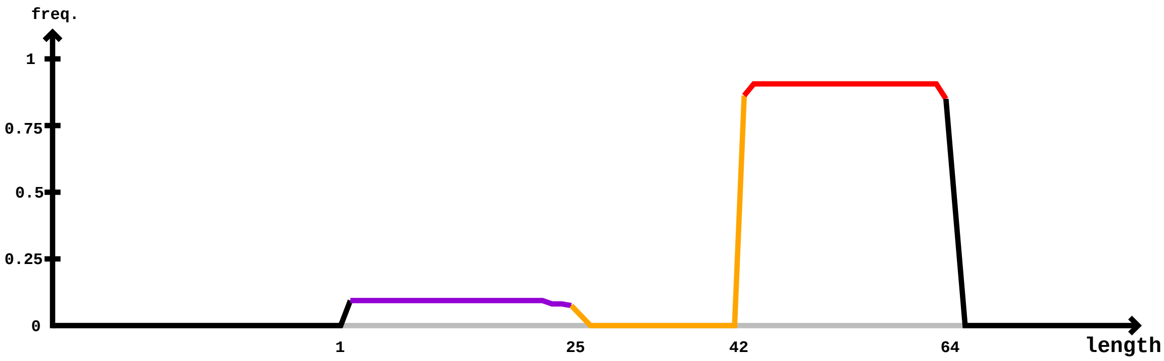

Star Mature

```
5'- ccgccccagagagcacugagggccagauacuguaaacacccuacacucucagcugugcacagugagaaagcugggaggaggcuguuuacucucucugccuuggaagucaac   -3'   obs
    ccgccccagagagcacugagggccagauacuguaaacacccuacacucucagcugugcacagugagaaagcugggaggaggcuguuuacucucucugccuuggaagucaac         exp
    ..(((((((......))).))))((((....(((((((.(((.(.(((((((((.............))))))))))))).)))))))....))))...............    reads   mm   sample
    .............................cuguaaacacccuacacucuc.............................................................    2       0    seq
    .............................cuguaaacacccuacacucucag...........................................................    1       0    seq
    .............................cuguaaacacccuacacucucagc..........................................................    7       0    seq
    .............................cuguaaacacccuacacucucagcu.........................................................    5       0    seq
    ......................................................................cugggaggaggcuguuuacuc....................    9       0    seq
    ......................................................................cugggaggaggcuguuuacucu...................    127     0    seq
    ......................................................................cugggaggaggcuguuuacucuc..................    2       0    seq
    .......................................................................ugggaggaggcuguuuacucu...................    6       0    seq
    .......................................................................ugggaggaggcuguuuacucuc..................    1       0    seq
```
